# Bioinformatics analysis of rabbit haemorrhagic disease virus genome

**DOI:** 10.1186/1743-422X-8-494

**Published:** 2011-11-01

**Authors:** Xiao-ting Tian, Bao-yu Li, Liang Zhang, Wen-qiang Jiao, Ji-xing Liu

**Affiliations:** 1 State Key Laboratory of Veterinary Etiological Biology, Key Laboratory of Grazing Animal Diseases of Ministry of Agriculture, Key Laboratory of Animal Virology of Ministry of Agriculture, State Key Laboratory of Veterinary Etiological Biology, Lanzhou Veterinary Research Institute, Chinese Academy of Agricultural Sciences, Xujia ping 1, Yanchang bu, Lanzhou, Gansu, Post Code 730046, China

**Keywords:** Rabbit haemorrhagic disease virus (RHDV), Codon usage, Evolution, Expression

## Abstract

**Background:**

Rabbit haemorrhagic disease virus (RHDV), as the pathogeny of Rabbit haemorrhagic disease, can cause a highly infectious and often fatal disease only affecting wild and domestic rabbits. Recent researches revealed that it, as one number of the Caliciviridae, has some specialties in its genome, its reproduction and so on.

**Results:**

In this report, we firstly analyzed its genome and two open reading frameworks (ORFs) from this aspect of codon usage bias. Our researches indicated that mutation pressure rather than natural is the most important determinant in RHDV with high codon bias, and the codon usage bias is nearly contrary between ORF1 and ORF2, which is maybe one of factors regulating the expression of VP60 (encoding by ORF1) and VP10 (encoding by ORF2). Furthermore, negative selective constraints on the RHDV whole genome implied that VP10 played an important role in RHDV lifecycle.

**Conclusions:**

We conjectured that VP10 might be beneficial for the replication, release or both of virus by inducing infected cell apoptosis initiate by RHDV. According to the results of the principal component analysis for ORF2 of RSCU, we firstly separated 30 RHDV into two genotypes, and the ENC values indicated ORF1 and ORF2 were independent among the evolution of RHDV.

## 1. Background

Synonymous codons are not used randomly [1]. The variation of codon usage among ORFs in different organisms is accounted by mutational pressure and translational selection as two main factors [2,3]. Levels and causes of codon usage bias are available to understand viral evolution and the interplay between viruses and the immune response [4]. Thus, many organisms such as bacteria, yeast, Drosophila, and mammals, have been studied in great detail up on codon usage bias and nucleotide composition [5]. However, same researches in viruses, especially in animal viruses, have been less studied. It has been observed that codon usage bias in human RNA viruses is related to mutational pressure, G+C content, the segmented nature of the genome and the route of transmission of the virus [6]. For some vertebrate DNA viruses, genome-wide mutational pressure is regarded as the main determinant of codon usage rather than natural selection for specific coding triplets [4]. Analysis of the bovine papillomavirus type 1 (BPV1) late genes has revealed a relationship between codon usage and tRNA availability [7]. In the mammalian papillomaviruses, it has been proposed that differences from the average codon usage frequencies in the host genome strongly influence both viral replication and gene expression [8]. Codon usage may play a key role in regulating latent versus productive infection in Epstein-Barr virus [9]. Recently, it was reported that codon usage is an important driving force in the evolution of astroviruses and small DNA viruses [10,11]. Clearly, studies of synonymous codon usage in viruses can reveal much about the molecular evolution of viruses or individual genes. Such information would be relevant in understanding the regulation of viral gene expression.

Up to now, little codon usage analysis has been performed on Rabbit haemorrhagic disease virus (RHDV), which is the pathogen causing Rabbit haemorrhagic disease (RHD), also known as rabbit calicivirus disease (RCD) or viral haemorrhagic disease (VHD), a highly infectious and often fatal disease that affects wild and domestic rabbits. Although the virus infects only rabbits, RHD continues to cause serious problems in different parts of the world. RHDV is a single positive stranded RNA virus without envelope, which contains two open reading frames (ORFs) separately encoding a predicted polyprotein and a minor structural protein named VP10 [12]. After the hydrolysis of self-coding 3C-like cysteinase, the polyprotein was finally hydrolyzed into 8 cleavage products including 7 nonstructural proteins and 1 structural protein named as VP60 [13,14]. Studies on the phylogenetic relationship of RHDVs showed only one serotype had been isolated, and no genotyping for RHDV was reported. It reported that the VP10 was translated with an efficiency of 20% of the preceding ORF1 [15]. In order to better understand the characteristics of the RHDV genome and to reveal more information about the viral genome, we have analyzed the codon usage and dinucleotide composition. In this report, we sought to address the following issues concerning codon usage in RHDV: (i) the extent and causes of codon bias in RHDV; (ii) A possible genotyping of RHDV; (iii) Codon usage bias as a factor reducing the expression of VP10 and (iiii) the evolution of the ORFs.

## 2. Materials and methods

### 2.1 Sequences

The 30 available complete RNA sequences of RHDV were obtained from GenBank randomly in January 2011. The serial number (SN), collection dates, isolated areas and GenBank accession numbers are listed in Table 1.

**Table 1:** Information of RHDV genomes

*SN*	*Strain*	*Isolation*	*Date*	**Accession No**.
1	UT-01	USA:Utah	2001	EU003582.1
2	NY-01	USA: New York	2001	EU003581.1
3	Italy-90	Italy	1990	EU003579.1
4	IN-05	USA: Indiana	2005	EU003578.1
5	NJ-2009	China: Nanjing	2009	HM623309.1
6	Iowa2000	USA: Iowa	2000	AF258618.2
7	pJG-RHDV-DD06	Ramsay Island	2007	EF363035.1
8	Bahrain	Bahrain	2006	DQ189077.1
9	CD/China	Changchun, China	2004	AY523410.1
10	RHDV-V351	Czech	1996	U54983.1
11	RHDV-Hokkaido	Japan	2002	AB300693.2
12	RHDV-FRG	Germany	1991	NC_001543.1
13	Meiningen	Germany	2007	EF558577.1
14	Jena	Germany	2007	EF558576.1
15	Hartmannsdorf	Germany	2007	EF558586.1
16	Rossi	Germany	2007	EF558584.1
17	Triptis	Germany	2007	EF558583.1
18	Dachswald	Germany	2007	EF558582.1
19	Erfurt	Germany	2007	EF558581.1
20	NZ61	New Zealand	2007	EF558580.1
21	NZ54	New Zealand	2007	EF558579.1
22	Eisenhuttenstadt	Germany	2007	EF558578.1
23	Ascot	United Kingdom	2007	EF558575.1
24	Wika	Germany	2007	EF558574.1
25	Frankfurt5	Germany	2007	EF558573.1
26	Frankfurt12	Germany	2007	EF558572.1
27	WHNRH	China	2005	DQ280493.1
28	BS89	Italy	1995	X87607.1
29	RHDV-SD	France	1993	Z29514.1
30	M67473.1	Germany	1991	M67473.1

### 2.2 The relative synonymous codon usage (RSCU) in RHDV

To investigate the characteristics of synonymous codon usage without the influence of amino acid composition, RSCU values of each codon in a ORF of RHDV were calculated according to previous reports (2 Sharp, Tuohy et al. 1986) as the followed formula:

$$\text{RSCU}=\frac{g_{ij}}{\overset{n_{i}}{\underset{j}{\sum }}g_{\;ij}}n_{i}$$

Where g_ij _is the observed number of the *i*th codon for *j*th amino acid which has n_i _type of synonymous codons. The codons with RSCU value higher than 1.0 have positive codon usage bias, while codons with value lower than 1.0 has relative negative codon usage bias. As RSCU values of some codons are nearly equal to 1.0, it means that these codons are chosen equally and randomly.

### 2.3 The content of each nucleotides and G+C at the synonymous third codon position (GC3s)

The index GC3s means the fraction of the nucleotides G+C at the synonymous third codon position, excluding Met, Trp, and the termination codons.

### 2.4 The effective number of codons (ENC)

The ENC, as the best estimator of absolute synonymous codon usage bias [16], was calculated for the quantification of the codon usage bias of each ORF [17]. The predicted values of ENC were calculated as

$$\text{ENC}=2+s+\frac{29}{s^{2}+\left(1-s^{2}\right)}$$

where s represents the given (G+C)_3_% value. The values of ENC can also be obtained by EMBOSS CHIPS program [18].

### 2.5 Dn and ds of two ORFs

Analyses were conducted with the Nei-Gojobori model [19], involving 30 nucleotide sequences. All positions containing gaps and missing data were eliminated. The values of dn, ds and ω (dn/ds) were calculated in MEGA4.0 [20].

### 2.6 Correspondence analysis (COA)

Multivariate statistical analysis can be used to explore the relationships between variables and samples. In this study, correspondence analysis was used to investigate the major trend in codon usage variation among ORFs. In this study, the complete coding region of each ORF was represented as a 59 dimensional vector, and each dimension corresponds to the RSCU value of one sense codon (excluding Met, Trp, and the termination codons) [21].

### 2.7 Correlation analysis

Correlation analysis was used to identify the relationship between nucleotide composition and synonymous codon usage pattern [22]. This analysis was implemented based on the Spearman's rank correlation analysis way.

All statistical processes were carried out by with statistical software SPSS 17.0 for windows.

## 3. Results

### 3.1 Measures of relative synonymous codon usage

The values of nucleotide contents in complete coding region of all 30 RHDV genomes were analyzed and listed in Table 2 and Table 3. Evidently, (C+G)% content of the ORF1 fluctuated from 50.889 to 51.557 with a mean value of 51.14557, and (C+G)% content of the ORF2 were ranged from 35.593 to 40.113 with a mean value of 37.6624, which were indicating that nucleotides A and U were the major elements of ORF2 against ORF1. Comparing the values of A_3_%, U_3_%, C_3_% and G_3_%, it is clear that C_3_% was distinctly high and A_3_% was the lowest of all in ORF1 of RHDV, while U_3_% was distinctly high and C_3_% was the lowest of all in ORF2 of RHDV. The (C_3_+G_3_) % in ORF1 fluctuated from 57.014 to 58.977 with a mean value of 57.68287 and (C_3_+G_3_)% were range from 31.356 to 39.831 with a mean value of 34.8337. And the ENC values of ORF1 fluctuated from 54.192 to 55.491 with a mean value of 54.95 and ENC values of ORF2 displayed a far-ranging distribution from 39.771 to 51.964 with a mean value of 44.46. The ENC values of ORF1 were a little high indicating that there is a particular extent of codon preference in ORF1, but the codon usage is relatively randomly selected in ORF2 on the base of ENC values. The details of the overall relative synonymous codon usage (RSCU) values of 59 codons for each ORF in 30 RHDV genomes were listed in Table 4. Most preferentially used codons in ORF1 were C-ended or G-ended codons except Ala, Pro and Ser, however, A-ended or G-ended codons were preferred as the content of ORF2.

**Table 2:** Identified nucleotide contents in complete coding region (length > 250 bps) in the ORF1 of RHDV (30 isolates) genome

SN	A%	A_3_%	U%	U_3_%	C%	C_3_%	G%	G_3_%	(C+G)%	(C_3_+G_3_)%	ENC
1	25.302	18.252	23.340	23.497	25.544	33.348	25.814	24.904	51.358	58.252	54.786
2	25.387	18.294	23.738	24.691	25.146	32.281	25.729	24.733	51.386	57.014	55.201
3	25.515	18.678	23.298	23.795	25.657	33.220	25.529	24.307	51.186	57.527	55.05
4	25.899	19.488	22.758	21.876	26.141	35.053	25.203	23.582	51.344	58.635	54.68
5	25.515	18.593	23.554	24.136	25.373	32.878	25.558	24.392	50.931	57.270	55.491
6	25.458	18.294	23.554	24.222	25.444	32.921	25.544	24.563	50.988	57.484	55.268
7	25.359	18.806	23.454	23.667	25.487	33.262	25.700	24.264	51.187	57.526	54.723
8	25.402	18.721	23.412	23.625	25.544	33.305	25.643	24.350	51.187	57.655	55.031
9	25.615	19.062	23.383	23.625	25.544	33.433	25.458	23.881	51.002	57.314	54.906
10	25.430	18.593	23.383	23.966	25.629	33.006	25.558	24.435	51.187	57.441	55.439
11	25.288	17.910	23.596	24.435	25.402	32.751	25.714	24.904	51.116	57.665	54.984
12	25.529	18.635	23.412	23.838	25.515	33.092	25.544	24.435	51.059	57.527	55.203
13	25.387	18.380	23.611	23.966	25.316	33.006	25.686	24.648	51.002	57.654	54.681
14	25.274	18.124	23.426	23.582	25.544	33.433	25.757	24.861	51.301	58.294	54.548
15	25.203	18.166	23.724	24.691	25.188	32.239	25.885	24.904	51.073	57.143	55.429
16	25.487	18.721	23.326	23.326	25.601	33.603	25.586	24.350	51.187	57.953	55.148
17	25.444	18.507	23.369	23.582	25.572	33.433	25.615	24.478	51.187	57.911	55.27
18	25.572	18.806	23.539	24.179	25.416	32.836	25.473	24.179	50.889	57.015	55.417
19	25.487	18.507	23.582	24.136	25.359	32.964	25.572	24.392	50.931	57.356	55.384
20	25.558	18.806	23.426	23.966	25.473	32.878	25.544	24.350	51.017	57.228	55.165
21	25.544	18.721	23.426	24.009	25.529	33.006	25.501	24.264	51.030	57.270	55.156
22	25.160	17.783	23.312	23.326	25.729	33.689	25.800	25.203	51.529	58.892	54.682
23	25.487	18.806	23.511	23.710	25.529	33.433	25.473	24.051	51.002	57.487	54.192
24	25.387	18.593	23.497	23.667	25.572	33.348	25.544	24.392	51.116	57.740	54.213
25	25.330	18.635	23.483	23.582	25.615	33.433	25.572	24.350	51.187	57.783	54.238
26	25.387	18.593	23.511	23.710	25.572	33.390	25.529	24.307	51.101	57.697	54.285
27	25.330	18.209	23.511	24.264	25.487	32.964	25.672	24.563	51.159	57.527	55.267
28	25.448	18.643	23.443	23.635	25.576	33.362	25.533	24.360	51.109	57.722	54.614
29	25.174	17.868	23.269	23.156	25.686	33.817	25.871	25.160	51.557	58.977	54.842
30	25.529	18.635	23.412	23.838	25.515	33.092	25.544	24.435	51.059	57.527	55.203

**Table 3:** Identified nucleotide contents in complete coding region (length > 250 bps) in the ORF2 of RHDV (30 isolates) genome

SN	A%	A_3_%	U%	U_3_%	C%	C_3_%	G%	G_3_%	(C+G)%	(C_3_+G_3_)%	ENC
1	29.944	17.797	30.791	44.068	13.842	16.102	25.424	22.034	39.266	38.136	49.377
2	29.944	18.644	30.226	43.220	14.407	16.949	25.424	21.186	39.831	38.135	48.182
3	31.356	20.339	31.638	46.610	12.994	13.559	24.011	19.492	37.005	33.051	44.567
4	30.508	18.644	30.791	44.915	13.842	15.254	24.859	21.186	38.701	36.440	46.686
5	29.944	17.797	31.921	46.610	12.712	13.559	25.424	22.034	38.136	35.593	41.215
6	30.226	16.949	30.226	43.220	14.407	16.949	25.141	22.881	39.548	39.830	51.964
7	31.356	19.492	30.791	45.763	14.124	15.254	23.729	19.492	37.853	34.764	45.757
8	30.226	16.949	29.661	43.220	15.254	17.797	24.859	22.034	40.113	39.831	47.242
9	30.508	18.644	31.356	45.763	13.277	14.407	24.859	21.186	38.136	35.593	43.017
10	31.356	20.339	31.638	46.610	12.994	13.559	24.011	19.492	37.005	33.051	44.576
11	29.782	17.518	33.898	48.175	12.107	13.139	24.213	21.168	36.320	34.307	43.088
12	31.638	21.186	31.073	45.763	12.994	13.559	24.294	19.492	37.288	33.051	44.997
13	31.073	18.644	31.638	46.610	13.277	14.407	24.011	20.339	37.288	34.746	43.213
14	31.638	19.492	31.921	47.458	12.994	13.559	23.446	19.492	36.440	33.051	47.214
15	31.921	20.339	31.921	46.610	12.712	13.559	23.446	19.492	36.158	33.051	41.964
16	30.226	18.644	30.508	43.220	14.124	16.949	25.141	21.186	39.265	38.135	47.603
17	30.508	19.492	30.508	43.220	13.559	15.254	25.424	22.034	38.983	37.288	47.615
18	29.096	16.102	31.356	45.763	13.277	14.407	26.271	23.729	39.548	38.136	44.343
19	30.226	19.492	31.073	44.915	13.559	15.254	25.141	20.339	38.700	35.593	46.768
20	31.638	19.492	32.768	49.153	11.864	11.017	23.729	20.339	35.593	31.356	39.771
21	31.638	19.492	32.768	49.153	11.864	11.017	23.729	20.339	35.593	31.356	39.771
22	31.073	19.492	31.356	45.763	12.994	13.559	24.576	21.186	37.570	34.745	43.282
23	31.356	19.492	31.921	47.458	12.994	13.559	23.729	19.492	36.723	33.051	42.633
24	31.638	20.339	31.921	47.458	12.994	13.559	23.446	18.644	36.440	32.203	42.157
25	31.638	20.339	32.203	48.305	12.712	12.712	23.446	18.644	36.185	31.356	40.006
26	31.638	20.339	32.203	48.305	12.712	12.712	23.446	18.644	36.185	31.356	40.006
27	30.226	17.797	31.073	44.915	13.559	15.254	25.141	22.034	38.700	37.288	42.799
28	31.356	18.644	31.356	45.763	13.559	15.254	23.729	20.339	37.288	35.593	45.413
29	31.638	21.186	31.638	46.610	12.712	12.712	24.011	19.492	36.723	32.204	43.618
30	31.638	21.186	31.073	45.763	12.994	13.559	24.294	19.492	37.288	32.721	44.997

**Table 4:** Synonymous codon usage of the whole coding sequence in RHDV

AA^a^	Codon	RSCU in ORF1	RSCU in ORF2	AA^a^	Codon	RSCU in ORF1	RSCU in ORF2
Ala	GCA	1.238761	0.877698	Leu	CUA	0.582651	0.410596
	GCC	1.224431	1.165468		CUC	1.349825	0.397351
	GCG	0.567437	0.014388		CUG	1.188367	0.900662
	GCU	0.969371	1.942446		CUU	1.107137	0.821192
Arg	AGA	1.266604	1.481013		UUA	0.498412	1.350993
	AGG	2.026193	3.341772		UUG	1.273609	2.119205
	CGA	0.303087	0	Lys	AAA	0.699282	0.837209
	CGC	0.991581	1.177215		AAG	1.300718	1.162791
	CGG	0.445276	0	Phe	UUC	0.909962	0.360902
	CGU	0.967259	0		UUU	1.090038	1.639098
Asn	AAC	1.562517	0.140845	Pro	CCA	1.370342	2
	AAU	0.437483	1.859155		CCC	1.204832	0.451613
Asp	GAC	1.576108	0.909091		CCG	0.45541	0
	GAU	0.423892	1.090909		CCU	0.969417	1.548387
Cys	UGC	1.034803	0	Ser	AGC	0.969041	1.567416
	UGU	0.965197	0		AGU	1.104135	3.370787
Gln	CAA	0.798416	1.651613		UCA	1.437974	0
	CAG	1.201584	0.348387		UCC	1.226239	0.522472
Glu	GAA	0.843523	0.8		UCG	0.558562	0
	GAG	1.156477	1.2		UCU	0.704048	0.539326
Gly	GGA	0.669081	0.797508	Ile	AUA	0.574538	0
	GGC	1.262976	0.984424		AUC	1.247451	0.525
	GGG	0.944991	0.398754		AUU	1.17801	2.475
	GGU	1.122952	1.819315	Tyr	UAC	1.285714	0.086022
His	CAC	1.412429	0		UAU	0.714286	1.913978
	CAU	0.587571	2	Val	GUA	0.316211	0.763077
Thr	ACA	1.212516	0.129032		GUC	1.050408	0.258462
	ACC	1.379635	2		GUG	1.163066	0.615385
	ACG	0.496292	0		GUU	1.470315	2.363077
	ACU	0.911557	1.870968				

In addition, the dn, ds and ω(dN/dS) values of ORF1 were separately 0.014, 0.338 and 0.041, and the values of ORF2 were 0.034, 0.103 and 0.034, respectively. The ω values of two ORFs in RHDV genome are generally low, indicating that the RHDV whole genome is subject to relatively strong selective constraints.

### 3.2 Correspondence analysis

**Figure 1:**
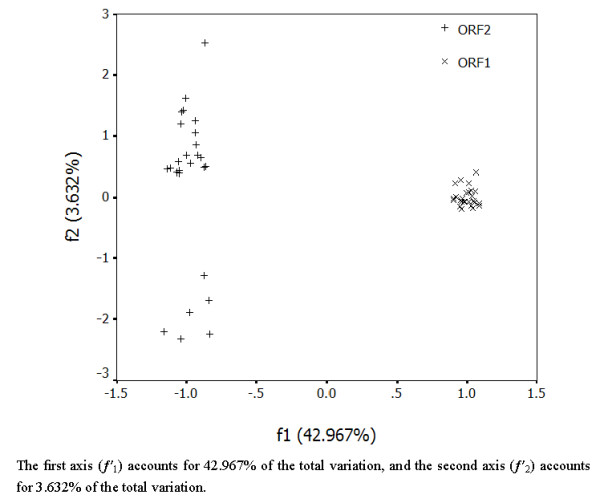
**A plot of value of the first and second axis of RHDV genome in COA**. The first axis (*f*'_1_) accounts for 42.967% of the total variation, and the second axis (*f*'_2_) accounts for 3.632% of the total variation.

COA was used to investigate the major trend in codon usage variation between two ORFs of all 30 RHDV selected for this study. After COA for RHDV Genome, one major trend in the first axis (*f*'_1_) which accounted for 42.967% of the total variation, and another major trend in the second axis (*f*'_2_) which accounted for 3.632% of the total variation. The coordinate of the complete coding region of each ORF was plotted in Figure 1 defining by the first and second principal axes. It is clear that coordinate of each ORF is relatively isolated. Interestingly, we found that relatively isolated spots from ORF2 tend to cluster into two groups: the ordinate value of one group (marked as Group 1) is positive value and the other one (marked as Group 2) is negative value. Interestingly, all of those strains isolated before 2000 belonged to Group 2.

### 3.3 Correlation analysis

To estimate whether the evolution of RHDV genome on codon usage was regulated by mutation pressure or natural selection, the A%, U%, C%, G% and (C+G)% were compared with A_3_%, U_3_%, C_3_%, G_3_% and (C_3_+G_3_)%, respectively (Table 5). There is a complex correlation among nucleotide compositions. In detail, A_3_%, U_3_%, C_3_% and G_3_% have a significant negative correlation with G%, C%, U% and A% and positive correlation with A%, U%, C% and G%, respectively. It suggests that nucleotide constraint may influence synonymous codon usage patterns. However, A_3_% has non-correlation with U% and C%, and U_3_% has non-correlation with A% and G%, respectively, which haven't indicated any peculiarity about synonymous codon usage. Furthermore, C_3_% and G_3_% have non-correlation with A%, G% and U%, C%, respectively, indicating these data don't reflect the true feature of synonymous codon usage as well. Therefore, linear regression analysis was implemented to analyze the correlation between synonymous codon usage bias and nucleotide compositions. Details of correlation analysis between the first two principle axes (*f*'_1 _and *f*'_2_) of each RHDV genome in COA and nucleotide contents were listed in Table 6. In surprise, only f2 values are closely related to base nucleotide A and G content on the third codon position only, suggesting that nucleotide A and G is a factor influencing the synonymous codon usage pattern of RHDV genome. However, *f*'_1 _value has non-correlation with base nucleotide contents on the third codon position; it is observably suggest that codon usage patterns in RHDV were probably influenced by other factors, such as the second structure of viral genome and limits of host. In spite of that, compositional constraint is a factor shaping the pattern of synonymous codon usage in RHDV genome.

**Table 5:** Summary of correlation analysis between the A, U, C, G contents and A_3_, U_3_, C_3_, G_3 _contents in all selected samples

	A_3_%	U_3_%	C_3_%	G_3_%	(C_3_+G_3_)%
A%	r = 0.869**	r = -0.340^NS^	r = -0.358^NS^	r = -0.865**	r = -0.266**
U%	r = -0.436^NS^	r = 0.921**	r = -0.902**	r = -0.366^NS^	r = -0.652**
C%	r = 0.376^NS^	r = -0.919**	r = 0.932**	r = -0.352^NS^	r = 0.692**
G%	r = -0.860**	r = -0.377^NS^	r = -0.437^NS^	r = 0.910**	r = 0.220**
(C+G)%	r = -0.331 ^NS^	r = -0.649**	r = 0.636**	r = 0.399*	r = 0.915**

^a^r value in this table is calculated in each correlation analysis.

NS means non-significant (p > 0.05).

* means 0.01 < p < 0.05

**means p < 0.01

**Table 6:** Summary of correlation analysis between the f1, f2 contents and A_3_, U_3_, C_3_, G_3_, C3+G3 contents in all selected samples

Base compositions	*f_1_' *(42.967%)	*f_2_' *(3.632%)
A_3_%	r = -0.051^NS^	r = -0.740**
U_3_%	r = 0.243^NS^	r = 0.314^NS^
C_3_%	r = -0.291^NS^	r = -0.298^NS^
G_3_%	r = 0.108^NS^	r = 0.723**
(C_3_+G_3_)%	r = -0.216^NS^	r = 0.205^NS^

^a^r value in this table is calculated in each correlation analysis.

NS means non-significant.

* means 0.01 < p < 0.05

**means p < 0.01

## 4. Discussion

There have been more and more features that are unique to RHDV within the family *Caliciviridae*, including its single host tropism, its genome and its VP10 as a structural protein with unknown function. After we analyzed synonymous codon usage in RHDV (Table 2), we obtained several conclusions and conjectures as followed.

### 4.1 Mutational bias as a main factor leading to synonymous codon usage variation

**Figure 2:**
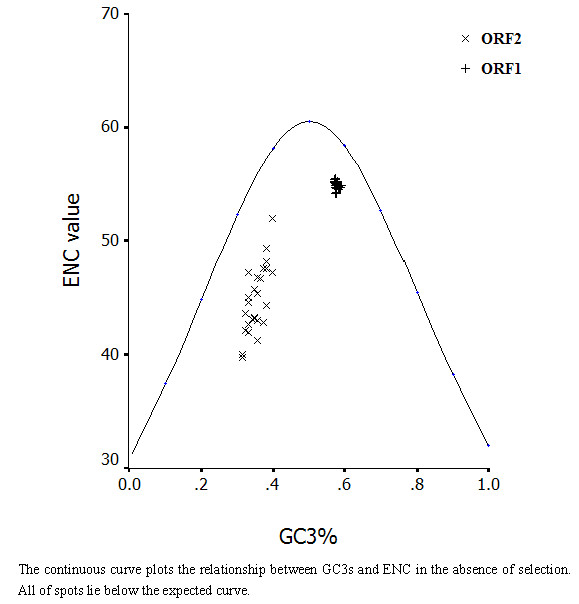
**Effective number of codons used in each ORF plotted against the GC3s**. The continuous curve plots the relationship between GC3s and ENC in the absence of selection. All of spots lie below the expected curve.

ENC-plot, as a general strategy, was utilized to investigate patterns of synonymous codon usage. The ENC-plots of ORFs constrained only by a C_3_+G_3 _composition will lie on or just below the curve of the predicted values [18]. ENC values of RHDV genomes were plotted against its corresponding (C_3_+G_3_) %. All of the spots lie below the curve of the predicted values, as shown in Figure 2, suggesting that the codon usage bias in all these 30 RHDV genomes is principally influenced by the mutational bias.

### 4.2 A proof for codon usage bias as a factor reducing the expression of VP10

As we know, the efficiency of gene expression is influenced by regulator sequences or elements and codon usage bias. It reported that the RNA sequence of the 3-terminal 84 nucleotides of ORF1were found to be crucial for VP10 expression instead of the encoded peptide. VP10 coding by ORF2 has been reported as a low expressive structural protein against VP60 coding by ORF1 [5]. And its efficiency of translation is only 20% of VP60. According to results showed by Table 4, it revealed the differences in codon usage patterns of two ORFs, which is a possible factor reducing the expression of VP10.

### 4.3 Negative selective constraints on the RHDV whole genome

Although VP10 encoded by ORF2, as a minor structural protein with unknown functions, has been described by LIU as a nonessential protein for virus infectivity, the ω value of ORF2 suggests VP10 plays an important role in the certain stage of whole RHDV lifecycle. After combining with low expression and ω value of VP10, we conjectured that VP10 might be beneficial for the replication, release or both of virus by inducing infected cell apoptosis initiate by RHDV. This mechanism has been confirmed in various positive-chain RNA viruses, including coxsackievirus, dengue virus, equine arterivirus, foot-and-mouth disease virus, hepatitis C virus, poliovirus, rhinovirus, and severe acute respiratory syndrome [23-29], although the details remain elusive.

### 4.4 Independent evolution of ORF1 and ORF2

As preceding description, ENC reflects the evolution of codon usage variation and nucleotide composition to some degree. After the correlation analysis of ENC values between ORF1 and ORF2 (Table 7), the related coefficient of ENC values of two ORFs is 0.230, and p value is 0.222 more than 0.05. These data revealed that no correlation existed in ENC values of two ORFs, indicating that codon usage patterns and evolution of two ORFs are separated each other. Further, this information maybe helps us well understand why RSCU and ENC between two ORFs are quite different.

**Table 7:** Summary of correlation analysis between ENC value of ORF1 and ENC value of ORF2

	ENC value of ORF1	ENC value of ORF2
ENC value of ORF1	r = 1, p = 0	r = 0.230, p = 0.222 > 0.05
ENC value of ORF2	r = 0.230, p = 0.222 > 0.05	r = 1, p = 0

### 4.5 A possible genotyping basis

Interestingly, we found that relatively isolated spots from ORF2 tend to cluster into two groups: the ordinate value of one group (marked as Group 1) is positive value and the other one (marked as Group 2) is negative value. And all of those strains isolated before 2000 belonged to Group 2, including *Italy-90, RHDV-V351, RHDV-FRG, BS89, RHDV-SD *and *M67473.1*. Although RHDV has been reported as only one type, this may be a reference on dividing into two genotypes.

## 5. Conclusion

In this report, we firstly analyzed its genome and two open reading frameworks (ORFs) from this aspect of codon usage bias. Our researches indicated that mutation pressure rather than natural is the most important determinant in RHDV with high codon bias, and the codon usage bias is nearly contrary between ORF1 and ORF2, which is maybe one of factors regulating the expression of VP60 (encoding by ORF1) and VP10 (encoding by ORF2). Furthermore, negative selective constraints on the RHDV whole genome implied that VP10 played an important role in RHDV lifecycle. We conjectured that VP10 might be beneficial for the replication, release or both of virus by inducing infected cell apoptosis initiate by RHDV. According to the results of the principal component analysis for ORF2 of RSCU, we firstly separated 30 RHDV into two genotypes, and the ENC values indicated ORF1 and ORF2 were independent among the evolution of RHDV. All the results will guide the next researches on the RHDV as a reference.

## Competing interests

The authors declare that they have no competing interests.

## Authors' contributions

XTT and BYL contributed equally to the original draft of the manuscript, and approved the final version. ZL and WQJ contributed to conception and design of the manuscript, and revised the manuscript. LJX is the corresponding author. All authors have read and approved the final manuscript.
